# Screen Time as a Determinant of Chosen Aspects of Lifestyle: A Cross-Sectional Study of 10- to 12-Year-Old Schoolchildren in Poland

**DOI:** 10.3390/nu17172891

**Published:** 2025-09-07

**Authors:** Joanna Myszkowska-Ryciak, Jadwiga Hamulka, Ewa Czarniecka-Skubina, Jerzy Gębski, Agata Chmurzynska, Krystyna Gutkowska

**Affiliations:** 1 Institute of Human Nutrition Sciences, Warsaw University of Life Sciences, 02-787 Warsaw, Poland; joanna_myszkowska-ryciak@sggw.edu.pl (J.M.-R.); ewa_czarniecka-skubina@sggw.edu.pl (E.C.-S.); jerzy_gebski@sggw.edu.pl (J.G.);; 2 Department of Human Nutrition and Dietetics, Poznań University of Life Sciences, 60-637 Poznan, Poland

**Keywords:** adolescents, lifestyle, electronic media exposure, diet, physical activity, weight status, family meals

## Abstract

**Objective:** The study aimed to analyze the relationship between screen time (ST) duration, body weight status (BWS), and selected lifestyle behaviors in children aged 10–12. **Methods:** A cross-sectional study of 7763 (50.8% girls) Polish schoolchildren was conducted in 2023–2024. Data on ST, physical activity (PA), sleep duration (SD), frequency of consumption of unhealthy foods, family meals (FM), and sociodemographic data were collected using a paper questionnaire. Anthropometric data were obtained from measurements; body mass index (BMI) was used to assess BWS, and the waist-to-height ratio to measure central obesity. A logistic regression model was performed to assess the effect of unhealthy food consumption, FM, BWS, PA level, and SD on the odds of excessive ST (>2 h/day). **Results:** Girls were less likely to extend ST than boys (OR: 0.78; 95% CI: 0.70–0.86). Increased PA had a limiting effect on the dependent variable (moderate OR: 0.64; 95% CI: 0.53–0.77; vigorous OR: 0.37; 95% CI: 0.31–0.45). Sleeping 6–8 h per day was associated with a 41.6% increase in prolonged ST (OR: 1.42; 95% CI: 1.27–1.57). Overweight/obese individuals were 39.6% more likely to exceed ST compared to normal-weight peers (OR: 1.40; 95% CI: 1.16–1.68). Living in a village and a smaller city increased the odds of excessive ST (OR: 1.12; 95% CI: 1.07–1.41 and OR: 1.18; 95% CI: 1.03–1.34). **Conclusions:** Excessive body mass and unhealthy dietary habits, particularly sugary beverages, have been identified as significant risk factors for excessive ST. Optimal SD, high PA, and regular FM might have a protective effect on ST. This knowledge will contribute to designing more tailored and effective educational interventions promoting healthy lifestyles in children.

## 1. Introduction

The advent of modern digital technologies has precipitated a substantial increase in the time people spend on electronic devices [1]. This phenomenon has escalated further during the COVID-19 pandemic, with the highest increase in total and leisure screen time (ST) among children [2,3] and adolescents [4]. During the COVID-19 pandemic, ST among individuals aged 12 to 18 years increased by an average of 110 min per day from pre-pandemic levels [4].

A substantial body of research has identified a negative relationship between the duration of ST in childhood/adolescence and subsequent outcomes. These outcomes include but are not limited to poor sleep quality [5,6,7], insufficient physical activity [7], impaired language and communication skills [8], mental and behavioral health concerns [9,10,11,12], and suboptimal academic performance, especially in the case of adolescents [13]. High ST compared with low ST was associated with a statistically significant 64% increased risk of metabolic syndrome among children and adolescents, with every 2 h of daily ST associated with a 29% increased risk of metabolic syndrome [14]. Children and adolescents who spend more time with digital devices demonstrate lower muscular strength, cardiorespiratory endurance (CRE), and fundamental movement skills [15]. However, active video games might improve locomotor and stability skills in the case of children with motor skill deficits caused by developmental disabilities, such as cerebral palsy and Down syndrome [16], as well as improve balance, postural stability, and agility among healthy children [17].

The health of children is influenced by a multitude of factors that frequently interact with each other. These factors include body weight status, eating habits, physical activity level, time spent in front of screens, and sleep duration [18,19,20,21]. An unhealthy diet, marked by high consumption of processed foods and sweetened beverages, is associated with the development of overweight and obesity [22,23,24]. These conditions, in turn, are often accompanied by an increase in time spent in front of screens. ST is associated with higher energy intake, particularly from high-calorie, low-nutrient foods advertised during screen activities [25], and lower sleep quality [5]. Conversely, reduced physical activity and inadequate sleep duration can further exacerbate this problem, creating a negative cycle in which children become increasingly inactive, which in turn promotes further weight gain [26,27]. The consumption of family meals has been demonstrated to be associated with a reduced risk of excess body weight [28] and enhanced dietary quality among children and adolescents [29]. However, it is important to note that family meals do not necessarily preclude children from using electronic media during meals [30], and family meals might not overcome the adverse impact on diet quality of having the TV on at mealtimes [31]. It is important to note that ST may be associated with different health indicators and behaviors, and their clustering should, therefore, be studied independently. Additionally, existing relationships may vary depending on factors such as age, sex, or place of residence.

The present article aims to examine the relationship between ST and weight status, unhealthy dietary habits, physical activity, and sleep duration among children, as well as consumption of family meals (FM). Furthermore, the objective of this study is to ascertain the impact of these covariates on the risk of excessive ST among schoolchildren. To the best of our knowledge, this is the only observation of a large group of children aged 10–12 linking ST with lifestyle behaviors and body weight status (BWS) in Poland. Comprehension of these interactions is imperative to formulating efficacious preventive strategies that could assist in reducing ST and consequently enhance children’s health. Particular attention should be directed toward children aged 10–12 years, as this is when hormonal changes associated with puberty begin, which affect, among other factors, body weight and composition [32,33]. Early adolescence is characterized by an escalating need for autonomy from parents and guardians, manifesting also in dietary choices and lifestyle behaviors strongly influenced by peers [34]. On the other hand, lifestyle behaviors (e.g., unhealthy diet and physical inactivity) are modifiable and tend to be established during childhood and young adulthood [35]. Thus, developing positive health habits during this period of life can yield significant health benefits in the future.

## 2. Materials and Methods

### 2.1. Participants and Setting

The present study focused on a sample of schoolchildren aged 10–12 from primary schools enrolled in the Junior-Edu-Żywienie (JEŻ) Project. The study was conducted between April 2022 and November 2023 in 2218 schools (15.1% of all primary schools in Poland), recruited from all voivodeships in Poland. The method of recruiting schools was intended to obtain a representative sample of children aged 7–12 with a school class as the smallest sampling unit. A comprehensive description of the project has been published in a previous paper [36,37].

A total of 7763 schoolchildren aged 10–12 years, for whom complete data were available, were eligible for this study.

### 2.2. Design and Data Collection

This study was conducted in the classes participating in the JEŻ Project. Schoolchildren were asked to complete a paper questionnaire during a classroom activity. Prior to completing the survey, they received instructions from the researcher, who was present throughout the entire time the schoolchildren were completing the questionnaire. The questionnaire was based on a validated tool designed for Polish teenagers, the SF-FFQ4PolishChildren [38], and was structured into five sections: nutrition-related knowledge, dietary habits, attitudes toward eating, selected aspects of lifestyle: physical activity, screen time (ST), and sleep duration (SD), as well as socio-economic issues. The completion of the questionnaire required approximately 30–45 min. Then, each questionnaire was assigned a code by researchers, and it was verified that all responses were documented. Anthropometric measurements were obtained from children whose parents provided consent, either before or after completing the questionnaire, in a designated room conducive to optimal performance.

### 2.3. Ethics Approval

The study protocol was approved by the Ethics Committee of the Institute of Human Nutrition Sciences at the Warsaw University of Life Sciences in Poland (Resolution No. 18/2022, 15 March 2022). The guidelines of the Declaration of Helsinki were followed during the study, and written informed consent was obtained from parents/caregivers for their children’s participation.

### 2.4. Screen Time

The assessment of ST was based on children’s self-reports, wherein they were permitted to select from six response options, ranging from “less than 2 h per day” to “10 or more hours per day,” with time intervals of two hours. Examples of ST behavior were provided (e.g., watching TV/video/DVD/PC/game consoles) for better understanding. The responses were then grouped into subgroups: “less than 2 h/day”, “2–4 h/day”, and “more than 4 h/day”. The value of 2 h was used as the cut-off point, under the recommendations of the American Academy of Pediatrics (AAP) [39] and the Polish Institute of Food and Nutrition [40]; any response that exceeded this threshold was categorized as high ST.

### 2.5. Unhealthy Dietary Patterns

The prevalence of unhealthy eating behaviors was evaluated by examining the frequency of consumption of fast foods, salty snacks, sweets, and sugar-sweetened beverages. Schoolchildren were instructed to select responses from the cafeteria of answers, with designated daily frequencies assigned to each option (“never or almost never”—0 times/day, “less than once a week”—0.06 times/day, “once a week”—0.14 times/day, “2–4 times/week”—0.43 times/day, “5–6 times/week”—0.79 times/day, “every day”—1 time/day, and “several times a day”—2 times/day) [38]. The consumption frequency of the examined foods was categorized as high (at least five times per week) or low (less than five times per week). Furthermore, the schoolchildren were queried on the frequency of their family meals (FM) with the possible responses: not at all; less than 1 time/week; 1–2 days/week; 3–4 days/week (these responses were grouped as low FM; 5–6 days/week; or every day—high FM, respectively).

### 2.6. Physical Activity

The habitual leisure-time physical activity of the children was assessed based on their self-reported responses, which were limited to three options: “low (more time spent sitting, watching TV, in front of a computer, reading, light housework, or a short walk up to 2 h a week), “moderate” (walking, cycling, gymnastics, working-out at home, or other light physical activity performed 2–3 h/week), or “vigorous” (cycling, running, working-out at home, or other sports activities requiring physical effort over 3 h/week). To facilitate informed decision-making, supplementary clarifications have been appended to each response option. A detailed description is available in the preceding publications [36,41].

### 2.7. Sleep Duration

The habitual duration of sleep (on both weekdays and weekends) was assessed based on the children’s self-report, who could choose the following response options: “less than 6 h per day,” “from 6 to almost 8 h per day,” or “8 or more hours per day.” The latter response was the cut-off point according to the average sleep duration recommended for the age groups represented in the present study [40].

### 2.8. Anthropometric Data

All measurements were conducted under the International Standards for Anthropometric Assessment (ISAK) recommendations by trained specialists using the same devices [42]. Body weight (BW, kg) was measured in light indoor clothing without shoes to the nearest 0.1 kg with an electronic digital scale (TANITA Corporation, Tokyo, Japan). Standing height (H, cm) was measured with a portable stadiometer (TANITA Corporation, Tokyo, Japan) with the head in a horizontal Frankfort plane position, barefoot, with a precision of 0.1 cm [43]. Waist (WC, cm) and hip circumferences (HC, cm) were measured with inelastic tape (SECA 201, Hamburg, Germany) with a resolution of 0.1 cm according to the standard procedure [44]. Based on obtained anthropometric data, age-specific and sex-specific BMI (Body Mass Index, kg/m^2^) and BMI z-scores were calculated to categorize children as underweight (BMI z-score < −1.0), normal weight (BMI z-score between −1.0 and 1.0), and overweight/obese (BMI z-score > 1.0) [45]. The waist-to-height ratio (WHtR) was calculated, with values ≥ 0.5 being indicative of central obesity [46].

### 2.9. Statistical Analyses

Analyses were conducted using SAS 9.4 (Statistical Analysis Software, SAS Institute Inc., Cary, NC, USA). Descriptive statistics were generated for all variables of interest. The normality of variable distribution was verified with the Kolmogorov–Smirnov test prior to the statistical analysis. The Mann–Whitney U-test was used to compare groups for continuous variables, and the chi-square test was used for categorical data analysis. A logistic regression model was performed to assess the impact of unhealthy food consumption, body weight status, physical activity level, and sleep duration (independent variables) on the ST duration (dependent variable). The model was adjusted for sex, age, and place of residence. The relationships between the qualitative variables were analyzed using multiple correspondence analysis. The analyses were adjusted for child sex, age, and place of residence, including the size of the locality (e.g., villages, towns with up to 100,000 inhabitants, and large urban agglomerations). A *p*-value of <0.05 was considered to indicate statistical significance.

## 3. Results

The study involved a total of 7763 schoolchildren (49.2% boys), of whom 3087 were 10 years old (38.17% boys), 2556 were 11 years old (33.17% boys), and 2120 were 12 years old (28.66% boys). The majority of the participants in the study resided in urban areas. The detailed characteristics of the study group in terms of the analyzed parameters are presented in Table 1. The majority of schoolchildren had a normal body weight, with a slightly lower percentage of those classified as underweight compared to those with excessive body weight. No significant differences in body weight status (BWS) were observed between boys and girls; however, central obesity was more prevalent among male subjects. Nearly half of the boys declared vigorous physical activity (PA), while moderate activity predominated among the girls. The study also examined sleep duration (SD), with half of the participants reporting a sleep duration of between six and eight hours per day. Boys were more likely than girls to achieve a minimum of eight hours of sleep. Regarding screen time (ST), approximately one-third of the schoolchildren spent less than two hours per day using electronic screens, with a higher proportion of girls. Girls were less likely than boys to report spending more than four hours per day in front of a screen. Significant differences were observed in the frequency of consumption of all product groups analyzed by sex, whereas the frequency of family meals (FM) consumption was not associated with sex.

A detailed insight into the relationship between adherence to ST recommendations (<2 h/day) and the examined determinants is presented in Table 2.

Significant differences were noted in the case of all the examined variables, namely sex, age, body weight status, WHtR, physical activity, sleep duration, place of residence, and family meals consumption, depending on the ST category. While all the examined determinants were statistically significant, the strength of the effect, as measured by Cramer’s V coefficient, indicates a weak correlation. The daily consumption frequency for all the assessed product groups was significantly higher for schoolchildren with ST exceeding two hours (*p* < 0.001). Detailed data regarding the analyzed parameters depending on compliance with screen time recommendations can be found in the (Supplementary Materials Table S1).

The next step in the analysis was to check whether and how the probability of exceeding ST recommendations depends on unhealthy food intake, FM, SD, PA intensity, and BWS (Table 3).

The model was adjusted for sex, age, and place of residence. Despite the statistical significance of these variables in the model, the values of the model parameters before adjustment remained unaffected. Adverse nutritional behaviors have been linked to increased ST duration. Specifically, a 1-level increase in the frequency of consumption increased the odds ratio of exceeding the ST recommendation in the case of fast foods by 11% (OR: 1.11; 95% CI: 1.05–1.17), sweet beverages by 19% (OR: 1.20; 95% CI: 1.1), sweets by 11% (OR: 1.11; 95% CI: 1.06–1.15), and salty snacks by 12% (OR: 1.13; 95% CI: 1.07–1.18). Eating FM showed a beneficial effect: a high meal frequency reduced the odds of exceeding ST by 33% (OR: 0.67; 95% CI: 0.61–0.74). Increasing activity from low to moderate reduced the odds of exceeding ST by 34% (OR: 0.64; 95% CI: 0.53–0.77), and changing from low to vigorous reduced these odds by 63% (OR: 0.37; 95% CI: 0.31–0.45). Sleeping 6–8 h a day was associated with a 42% increase in the likelihood of engaging in extended ST (OR: 1.42; 95% CI: 1.27–1.57) compared to individuals who sleep 8 h or more. Overweight/obese individuals exhibited a 40% higher likelihood of extended ST than individuals of normal weight (OR: 1.40; 95% CI: 1.16–1.68). The WHtR variable was not significantly associated with exceeding ST. Children residing in rural areas exhibited the highest probability of extended ST, with an odds ratio of 1.23 (95% CI: 1.07–1.41) compared to children in cities with populations exceeding 100,000. In contrast, children residing in cities with up to 100,000 inhabitants exhibited a 17% higher probability of engaging in long ST than those with more than 100,000 inhabitants. Girls were 22% less likely to engage in extended ST than boys (OR: 0.78; 95% CI: 0.70–0.86). Age was significantly associated with exceeding ST, with 11-year-olds demonstrating a 62% higher probability of spending more time on screens compared to 10-year-olds (OR: 1.62; 95% CI: 1.45–1). For 12-year-olds, the odds of ST ≥ 2 h increased by 169% (OR: 2.69; 95% CI: 2.36–3.06) compared to 10-year-olds.

**Figure 1:**
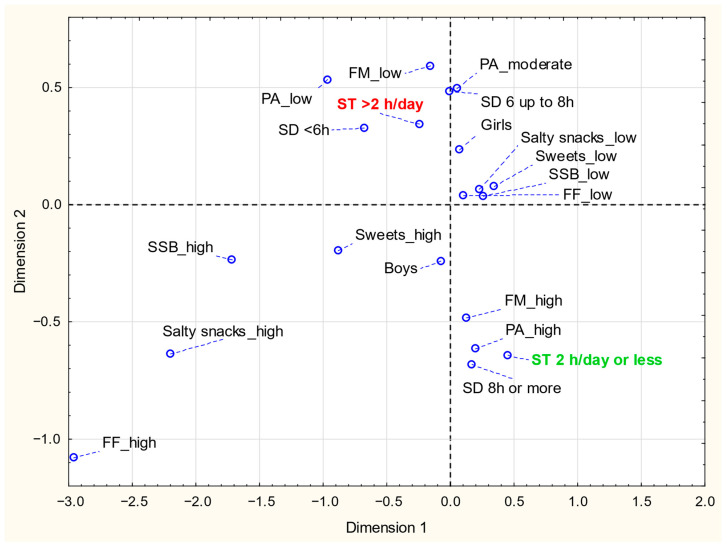
The results of the analysis of correspondence of examined variables: PA—physical activity (low, moderate, high); frequency of consumption (high—at least five times/week or low—less than five times/week) for: Salty snacks, FF—fast foods; SSB—sugar-sweetened beverages; Sweets; FM—family meals (low FM refers to 5–6 days/week; high FM refers to daily consumption; ST—screen time (2 h/d or less, >2 h/day); SD—sleep duration (<6 h, 6 up to 8 h, 8 h or more).

We further analyzed connections between the lifestyle behaviors, based on the correspondence analysis (Figure 1). Beneficial nutritional behaviors such as low frequency of fast food, chips and snacks, sweets, and sweetened beverages consumption were linked together. Conversely, unfavorable eating behaviors, such as a high frequency of the aforementioned foodstuffs, were related. High ST corresponded with the other adverse lifestyle behaviors: low physical activity, low FM frequency, and low sleep duration. Reduced ST was the opposite, associated with optimal sleep duration, high levels of physical activity, and regular family meals (at least 5 times/week).

## 4. Discussion

The majority of studies on screen time have focused on its association with weight status and physical activity [47,48,49,50,51]. The present study demonstrates an association between ST and various lifestyle behaviors in a large sample of adolescents aged 10–12 years. Identifying co-occurring effects can facilitate comprehending which behaviors should be considered simultaneously for health-promoting interventions, such as, e.g., health education programs.

Most examined adolescents (65%) exceeded the recommended ST, with boys demonstrating higher odds of such behavior. These data suggest a concerning trend of increasing ST among Poland’s adolescent population in recent years, as highlighted by a previous study conducted in 2015–2016 on a similar age group in Poland [52]. According to the study by Rocka et al. [53], 74% of children spent more than two hours using electronic devices for entertainment. However, the study period coincided with the COVID-19 pandemic, and the lockdown may explain this high result. Alarming trends are also observed in Western populations, where excessive ST concerns 40 to 80% of surveyed children and adolescents [54,55]. A substantial body of research has also demonstrated a positive correlation between age and ST in the pediatric population [53,56,57]. For Australian children transitioning from primary to secondary school, total ST increased by approximately 85.9 min per day over four years [57]. This phenomenon may be associated with the increased accessibility of electronic devices [58] and the diminishing parental oversight. In Poland, parents have designated 15 years as the age at which children can autonomously determine their screen time [53]. The present study offers further evidence to support the age-ST relationship, highlighting the pivotal age at which the change is most pronounced. Our findings suggest that educational initiatives designed to reduce ST in adolescents should be targeted at children aged 10 years or younger.

We confirmed sex as a significant determinant in adolescents’ ST habits. Boys generally report higher ST compared to girls [52,56,59]. Research indicates that boys tend to increase their ST significantly more than girls during adolescence [57]. They are more likely to play video games [60,61] and use ST as a coping mechanism for negative emotions [61]. Increased ST in boys is associated with behavioral problems, especially when it coincides with reduced physical activity [62]. At the same time, research indicates that boys’ ST strongly depends on their fathers’ ST [63], which may be an essential clue in designing preventive programs.

In our study, individuals with excess body weight had increased odds of exceeding ST. The relationship between excessive ST and overweightness/obesity in children and adolescents is well-documented, with multiple studies indicating a positive association [64,65]. However, the cause-and-effect relationship can be complex and multifaceted. Increased ST is associated with exposure to high-calorie, low-nutrient food and beverages [66] and higher energy intake, particularly from carbohydrates [25]. Conversely, overweight/obese children may prefer sedentary activities, including ST, due to physical discomfort or social factors. Additionally, a higher risk of depression in this group can lead to increased ST as a coping mechanism [67]. Consequently, further interventional and longitudinal studies are required to ascertain the causal relationships between these variables more precisely to tailor prevention programs better. 

In our study, the frequency of family meals was significantly related to the length of screen time, and the higher frequency of eating unhealthy food was associated with exceeding ST. A salient finding of our study is the demonstration of the potentially leading role of sugary beverages in increasing the risk of excessive screen time. A substantial body of research across diverse age groups has demonstrated an association between ST and unhealthy eating habits [53,56,68,69,70,71]. However, in this case, evaluating the direction of influence of both factors is challenging. On the one hand, observational studies suggest a higher propensity for snacking while using electronic devices [53]. The cross-sectional Spanish study revealed a correlation between ST exposure of more than one hour and increased frequency of sweets, fast food, and soft drink intake [71]. On the other hand, inadequate dietary habits may also be linked to a less healthy lifestyle [56], encompassing low physical activity levels and a tendency to engage in sedentary activities.

In the present study, an increase from low to moderate or vigorous physical activity was associated with a reduced likelihood of excessive ST. An inverse relationship between physical activity levels and ST has been previously demonstrated in the pediatric population in Poland [53]. However, a cross-sectional study of Canadian adolescents found no association between reported physical activity levels and the amount of time spent in screen-based sedentary behaviors [72]. Moreover, an analysis of European adolescents indicated an absence of any correlation between accelerometer-determined MVPA and the duration of television viewing [73]. Higher levels of physical activity might not reflect low levels of ST [74]. On the other hand, the use of electronic devices is increasingly being identified as a potentially beneficial behavior. To be precise, the use of Active Video Games (AVGs) by promoting additional physical activity [75] and increasing energy expenditure [76] has been reported as a potential tool for the maintenance and prevention of excess body weight in the pediatric population [77,78].

The relationship between sleep duration and ST remains unclear in the present study. A sleep duration slightly shorter than recommendations increased the odds of exceeding ST recommendations. However, such an association was not observed with less than 6 h of sleep duration. The relationship between sleep outcomes and ST in children and adolescents is well-documented, with numerous studies indicating a negative correlation between the two [5]. The lack of statistical significance in the case of <6 h/day sleep may be attributed to the relatively small number of children who indicated such an answer (less than 10%). Such an interpretation appears reasonable, particularly in light of the observation that a decrease in sleep duration to 6–8 h per day compared to >8 h/day resulted in 42% higher odds of excessive ST.

The present study found that residing in rural areas or smaller cities was associated with a higher likelihood of excessive ST compared to living in a metropolis. On the contrary, in a study by Nedjar-Guerre et al. [79], adolescents in urban areas tended to have higher ST compared to those in rural areas. The author explained this by the greater availability of screens and digital devices in urban settings. In Poland, the reduced ST observed among children residing in major urban centers may be attributable to the enhanced accessibility of alternative leisure activities, such as physical exercise facilities, swimming pools, and other recreational amenities. Such opportunities are often lacking in rural communities, and the climate in Poland for a significant period of the year is not conducive to outdoor activity. Conversely, a variety of electronic devices are both affordable and prevalent in rural areas and small towns. This finding suggests the necessity of educational initiatives aimed at reducing ST, in addition to the provision of suitable infrastructure that fosters engaging in off-screen activities for children and adolescents.

It is crucial to emphasize that the design of our study precludes the formulation of cause-and-effect conclusions. Nevertheless, the analysis of the variables studied suggests a close association between screen time and sleep duration, physical activity, and the consumption of family meals. Given the modifiability of these factors, it stands to reason that educational programs should consider them in unison to amplify their positive impact.

However, it is important to highlight the study’s strengths, including the large sample size, validated questionnaires, and assessing body mass status based on anthropometric measurements conducted by well-trained academic researchers—not self-reported data, which are subject to bias. The presence of researchers to clarify any queries during the questionnaire administration also enhances the reliability and accuracy of the responses collected.

This study is not without its limitations. Firstly, the cross-sectional design precludes the establishment of a causal relationship. Furthermore, the direction of the association is uncertain because decreased ST may be explained by healthy weight, more sleep, or more physical activity. Longitudinal studies are needed to determine whether increased ST directly leads to decreased sleep or excess weight in adolescents. Secondly, the reliance on self-reported data introduces potential biases related to social desirability and recall, which may compromise the accuracy of reported ST and associated parameters. Such biases may lead to systematic under- or overestimation of behaviors (e.g., underreporting of screen time, overreporting of physical activity), potentially distorting the observed associations. Future studies employing objective measures (e.g., accelerometers for physical activity and sleep, or digital tracking for screen time) would enhance the accuracy of such data by reducing bias and validating self-reported information. Thirdly, while the analysis incorporates various covariates, ST is influenced by numerous factors (e.g., the education level of parents or household income), and unadjusted variables may have influenced the observed results. Additionally, the sample was not randomly selected. Still, it reflects the sociodemographic status of Polish society, which the authors believe provides a solid foundation for creating such generalizations.

Despite these limitations, the study offers cross-sectional evidence of the association between ST and body weight, physical activity, sleep duration, and unhealthy eating behaviors among adolescents. The inclusion of multiple covariates, including sociodemographic and anthropometric variables, strengthens the consistency of the findings.

## 5. Conclusions

This study provides novel insights into a negative association between excessive screen time, excess body weight, and unhealthy lifestyle patterns among adolescents, indicating a significant age-related increase in the risk of excessive ST with age and that physical activity might be the strongest modifiable factor in limiting screen time. While an increase in unhealthy food consumption was associated with an elevated probability of increased ST, our study found that intake of sugary drinks had the most substantial effect. The present study also suggests a positive protective role of regular family meals and adequate sleep in limiting screen time, as well as a significant impact of the family environment on the ST patterns within this age group. We believe this knowledge will contribute to designing more tailored and effective educational interventions promoting healthy lifestyles.

## Figures and Tables

**Table 1:** Characteristics of the nutritional status and selected lifestyle behaviors by sex/sociodemographic variables.

Variables	Total N = 7763 (%)	Boys N = 3820 (%)	Girls N = 3943 (%)	*p*-Value
Body weight status category				
Underweight	888 (11.44)	433 (11.34)	455 (11.54)	0.955 ^A^
Normal weight	5687 (73.26)	2800 (73.30)	2887 (73.22)	
Overweight/obese	1188 (15.30)	587 (15.37)	601 (15.24)	
WHtR				
<0.5	6459 (83.20)	2934 (76.81)	3525 (89.40)	<0.001 ^A^
≥0.5	1304 (16.80)	886 (23.19)	418 (10.60)	
Physical activity level				
Low	895 (11.53)	491 (12.85)	404 (10.25)	<0.001 ^A^
Moderate	3376 (43.49)	1436 (37.59)	1940 (49.20)	
Vigorous	3492 (44.98)	1893 (49.55)	1599 (40.55)	
Sleep duration (hours/day)				
<6	756 (9.73)	355 (9.29)	401 (10.17)	<0.001 ^A^
6 up to 8	3885 (50.04)	1834 (48.01)	2051 (52.02)	
≥8	3122 (40.22)	1631 (42.70)	1491 (37.81)	
Screen time (hours/day)				
<2	2708 (34.88)	1217 (31.86)	1491 (37.81)	<0.001 ^A^
2 to 4	2852 (36.74)	1420 (37.17)	1432 (36.32)	
>4	2203 (28.38)	1183 (30.97)	1020 (25.87)	
Frequency of consumption (median value per day)				
Fast foods	0.06	0.06	0.06	<0.001 ^B^
Salty snacks	0.14	0.14	0.14	0.021 ^B^
Sweets	0.50	0.50	0.50	<0.001 ^B^
Sugar-sweetened beverages	0.14	0.14	0.14	<0.001 ^B^
Frequency of family meals				
Not at all	124 (3.2)	129 (6.1)	253 (3.2)	0.334 ^A^
Less than 1 time/week	205 (5.4)	241 (6.1)	446 (5.7)	
1–2 days/week	541 (14.2)	610 (15.5)	1151 (14.8)	
3–4 days/week	809 (21.2)	828 (21.0)	1637 (21.1)	
5–6 days/week	636 (16.6)	618 (15.7)	1254 (16.1)	
Every day	1505 (39.4)	1517 (38.5)	3022 (38.9)	
Place of residence				
Village	1651 (21.27)	810 (21.20)	841 (21.33)	0.066 ^A^
City ≤ 100,000 inhabitants	2923 (37.65)	1394 (36.49)	1529 (38.78)	
City > 100,000 inhabitants	3189 (41.08)	1616 (42.30)	1573 (39.89)	
Age (years)				
10	3087 (39.76)	1458 (38.17)	1629 (41.31)	0.007 ^A^
11	2556 (32.92)	1267 (33.17)	1289 (32.69)	
12	2120 (27.31)	1095 (28.66)	1025 (26.00)	

Note: ^A^—the chi-square test; ^B^—the Mann–Whitney U-test; estimates significant at the *p* < 0.05 level have been emboldened; WHtR—waist-to-height ratio; percentages may not add up to 100% due to rounding.

**Table 2:** The relationship between screen time and examined correlates.

Variables	X^2^ Statistics	Cramer’s V	*p*-Value
Sex	30.29	−0.06	<0.001
Age	260.01	0.18	<0.001
Body weight status	36.60	0.07	<0.001
WHtR	32.78	0.07	<0.001
Physical activity	211.67	0.17	<0.001
Sleep duration	86.34	0.11	<0.001
Place of residence	12.09	0.04	0.002
Family meals	103.39	−0.12	<0.001

Note: WHtR—the waist-to-height ratio.

**Table 3:** Logistic regression models on screen time (predicted: 2 h or more).

Variables	Estimate	Point Estimate OR	95% Wald Confidence Limits		*p*-Value ^A^
Intercept	−0.7133				<0.0001
Unhealthy food consumption frequency					
Fast foods	0.1022	1.108	1.045	1.173	0.001
Salty snacks	0.1177	1.125	1.07	1.183	<0.001
Sweets	0.1028	1.108	1.064	1.154	<0.001
Sugar-sweetened beverages	0.1779	1.195	1.147	1.244	<0.001
Family meals frequency (reference: low)					
High	−0.396	0.673	0.607	0.745	<0.001
Sleep duration (reference: ≥8 h)					
<6	−0.0636	0.938	0.785	1.121	0.484
6 up to 8	0.3476	1.416	1.274	1.573	<0.001
Physical activity level (reference: low)					
Moderate	−0.4534	0.635	0.526	0.768	<0.001
Vigorous	−0.9917	0.371	0.308	0.447	<0.001
Body weight status category (reference: normal body weight)					
Overweight/obese	0.3333	1.396	1.163	1.675	<0.001
Underweight	−0.0972	0.907	0.776	1.062	0.225
WHtR (reference: ≥0.5)					
<0.5	−0.1259	0.882	0.738	1.053	0.165
Place of residence (reference: cities > 100,000 inhabitants)					
Village	0.2059	1.229	1.073	1.407	0.003
City ≤ 100,000 inhabitants	0.1612	1.175	1.028	1.343	0.018
Sex (reference: boy)					
Girl	−0.2528	0.777	0.7	0.862	<0.001
Age/grade (reference: 10 years)					
11	0.4848	1.624	1.448	1.821	<0.001
12	0.9888	2.688	2.361	3.06	<0.001

Note: ^A^ the chi-square test; estimates significant at the *p* < 0.05 level have been emboldened; WHtR—waist-to-height ratio; low family meals frequency refers to 5–6 days/week; high family meals frequency refers to daily consumption.

## Data Availability

The datasets used for the study are available from the corresponding author upon reasonable request.

## Supplementary Materials

The following supporting information can be downloaded at: https://www.mdpi.com/article/10.3390/nu17172891/s1, Table S1: Characteristics of the nutritional status and selected lifestyle behaviors by adherence to ST recommendation; File S1: STROBE Statement—Checklist of items that should be included in reports of cross-sectional studies.

## Abbreviations

The following abbreviations are used in this manuscript:

95% CI	Confidence Intervals
BMI	Body Mass Index
BW	Body Weight
BWS	Body Weight Status
FF	Fast Foods
FM	Family Meals
HC	Hip Circumference
OR	Odds Ratio
PA	Physical Activity
SD	Sleep Duration
SS	Salty Snacks
SSB	Sugar-sweetened Beverages
ST	Screen Time
WC	Waist Circumference
WHtR	Waist-to-Height Ratio
