# Supplementary material for: Screen Time as a Determinant of Chosen Aspects of Lifestyle: A Cross-Sectional Study of 10- to 12-Year-Old Schoolchildren in Poland
Source: Nutrients. 2025 Sep 7;17(17):2891. doi: 10.3390/nu17172891 (PMC12430456; doi:10.3390/nu17172891)
Supplement: Supplementary file 1 [file nutrients-17-02891-s001.zip › nutrients-3842020 - supplementary material - file S1.pdf]

STROBE Statement—Checklist of items that should be included in reports of cross-sectional studies

Title: Screen time as a determinant of chosen aspects of lifestyle: a cross-sectional study of 10- to 12-year-old schoolchildren in Poland

	Item No	Recommendation	Page number
**Title and abstract**	1	(*a*) Indicate the study's design with a commonly used term in the title or the abstract	Cross-sectional study as stated in the Title and Abstract
		(*b*) Provide in the abstract an informative and balanced summary of what was done and what was found	Provided in Abstract
**Introduction**			
Background/rationale	2	Explain the scientific background and rationale for the investigation being reported	Included in the Background on pages 2, 3 and 4
Objectives	3	State specific objectives, including any prespecified hypotheses	Included in the Background on page 4
**Methods**			
Study design	4	Present key elements of study design early in the paper	Included in the Title and Abstract (cross-sectional study), and later in Methods (Participants and Settings and Design and Data Collection) on page 4
Setting	5	Describe the setting, locations, and relevant dates, including periods of recruitment, exposure, follow-up, and data collection	Described in Participants and Settings and Design and Data Collection
Participants	6	(*a*) Give the eligibility criteria, and the sources and methods of selection of participants	Described in Participants and Settings
Variables	7	Clearly define all outcomes, exposures, predictors, potential confounders, and effect modifiers. Give diagnostic criteria, if applicable	Described in Outcome: Screen Time, Unhealthy Dietary Patterns, Physical Activity, Sleep Duration, Anthropometric Data.
Data sources/ measurement	8*	For each variable of interest, give sources of data and details of methods of assessment (measurement). Describe comparability of assessment methods if there is more than one group	Described in Outcome: Screen Time, Unhealthy Dietary Patterns, Physical Activity, Sleep Duration, Anthropometric Data.
Bias	9	Describe any efforts to address potential sources of bias	Addressed in the limitations paragraph in the Discussion

Study size	10	Explain how the study size was arrived at	Not applicable
Quantitative variables	11	Explain how quantitative variables were handled in the analyses. If applicable, describe which groupings were chosen and why	Described in Potential Confounders and Statistical Analyses
Statistical methods	12	(*a*) Describe all statistical methods, including those used to control for confounding	Described in Statistical Analyses
		(*b*) Describe any methods used to examine subgroups and interactions	Described in Statistical Analyses
		(*c*) Explain how missing data were addressed	Not applicable – only participants with complete data were eligible for the study (explain in Participants and Settings)
		(*d*) If applicable, describe analytical methods taking account of sampling strategy	Described in Statistical Analyses
		(*e*) Describe any sensitivity analyses	Not applicable
**Results**			
Participants	13*	(a) Report numbers of individuals at each stage of study—eg numbers potentially eligible, examined for eligibility, confirmed eligible, included in the study, completing follow-up, and analysed	Described in Participants and setting and Results
		(b) Give reasons for non-participation at each stage	Not applicable
		(c) Consider use of a flow diagram	Not required
Descriptive data	14*	(a) Give characteristics of study participants (eg demographic, clinical, social) and information on exposures and potential confounders	Described in Results: Tables 1
		(b) Indicate number of participants with missing data for each variable of interest	Not applicable
Outcome data	15*	Report numbers of outcome events or summary measures	Not applicable
Main results	16	(*a*) Give unadjusted estimates and, if applicable, confounder-adjusted estimates and their precision (eg, 95% confidence interval). Make clear which confounders were adjusted for and why they were included	Described in Results: Tables 3
		(*b*) Report category boundaries when continuous variables were categorized	Described in Anthropometric Data
		(*c*) If relevant, consider translating estimates of relative risk into absolute risk for a meaningful time period	Not applicable
Other analyses	17	Report other analyses done—eg analyses of subgroups and interactions, and sensitivity analyses	Describe in Results, summarized in tables 1-3, S1, and figure 1
**Discussion**			

Key results	18	Summarise key results with reference to study objectives	Described in Results, summarized in Discussion
Limitations	19	Discuss limitations of the study, taking into account sources of potential bias or imprecision. Discuss both direction and magnitude of any potential bias	Addressed in Limitations
Interpretation	20	Give a cautious overall interpretation of results considering objectives, limitations, multiplicity of analyses, results from similar studies, and other relevant evidence	Described in Discussion
Generalisability	21	Discuss the generalisability (external validity) of the study results	Addressed in Discussion and Conclusions
**Other information**			
Funding	22	Give the source of funding and the role of the funders for the present study and, if applicable, for the original study on which the present article is based	Addressed in Founding

*Give information separately for exposed and unexposed groups.

Note: An Explanation and Elaboration article discusses each checklist item and gives methodological background and published examples of transparent reporting. The STROBE checklist is best used in conjunction with this article (freely available on the Web sites of PLoS Medicine at http://www.plosmedicine.org/, Annals of Internal Medicine at http://www.annals.org/, and Epidemiology at http://www.epidem.com/). Information on the STROBE Initiative is available at www.strobe-statement.org.
