# Supplementary material for: Screen Time as a Determinant of Chosen Aspects of Lifestyle: A Cross-Sectional Study of 10- to 12-Year-Old Schoolchildren in Poland
Source: Nutrients. 2025 Sep 7;17(17):2891. doi: 10.3390/nu17172891 (PMC12430456; doi:10.3390/nu17172891)
Supplement: Supplementary file 1 [file nutrients-17-02891-s001.zip › nutrients-3842020-supplementary - Table S1.pdf]

**Table S1.** Characteristics of the nutritional status and selected lifestyle behaviors by adherence to ST recommendation.

| **Variables** | **< 2 hours N = 2708 (%)** | **≥ 2 hours N = 5505 (%)** | **p-value** |
|---|---|---|---|
| Body weight status category | | | |
| Underweight | 334 (12.33) | 554 (10.96) | <0.001[A] |
| Normal weight | 2050 (75.70) | 3637 (71.95) | |
| Overweight / obese | 324 (11.96) | 864 (17.09) | |
| WHtR | | | |
| < 0.5 | 2343 (86.52) | 4116 (81.42) | <0.001[A] |
| ≥ 0.5 | 365 (13.48) | 939 (20.85) | |
| Physical activity level | | | |
| Low | 177 (6.54) | 718 (14.20) | <0.001[A] |
| Moderate | 1037 (38.29) | 2339 (46.27) | |
| Vigorous | 14.94 (55.17) | 1998 (39.53) | |
| Sleep duration (hours/day) | | | |
| < 6 | 274 (10.12) | 482 (9.54) | <0.001[A] |
| 6 up to 8 | 1166 (43.06) | 2719 (53.79) | |
| ≥ 8 | 1268 (46.82) | 1854 (36.68) | |
| Frequency of consumption (median value per day) | | | |
| Fast foods | 2 | 2 | <0.001[B] |
| Salty snacks | 3 | 3 | <0.001[B] |
| Sweets | 4 | 4 | <0.001[B] |
| Sugar-sweetened beverages | 2 | 2 | <0.001[B] |
| Frequency of family meals | | | |
| Not at all | 67 (2.50) | 186 (3.70) | <0.001[A] |
| Less than 1 time/week | 115 (4.20) | 331 (6.50) | |
| 1–2 days/week | 355 (13.10) | 796 (15.70) | |
| 3–4 days/week | 467 (17.20) | 1170 (23.10) | |
| 5–6 days/week | 431 (15.90) | 823 (16.30) | |
| Every day | 1273 (47.0) | 1749 (34.60) | |
| Place of residence | | | |
| Village | 597 (22.05) | 1054 (20.85) | 0.002[A] |
| City ≤ 100,000 inhabitants | 949 (35.04) | 1974 (39.05) | |
| City > 100,000 inhabitants | 1162 (42.91) | 2027 (40.10) | |
| Age (years) | | | |
| 10 | 1374 (50.74) | 1713 (33.89) | <0.001[A] |
| 11 | 845 (31.20) | 1711 (33.85) | |
| 12 | 489 (18.06) | 1631 (32.27) | |
| Sex | | | |
| Boys | 1217 (44.94) | 2603 (51.49) | <0.001[A] |
| Girls | 1491 (55.06) | 2452 (48.51) | |

Note: [A] – the chi square test; [B] - the Mann–Whitney U-test; estimates significant at the $p < 0.05$ level have been emboldened; percentages may not add up to 100% due to rounding.
